# Comparative Analysis of Human Adipose-Derived Mesenchymal Stem Cells from Orbital and Abdominal Fat

**DOI:** 10.1155/2018/3932615

**Published:** 2018-08-19

**Authors:** Sarmila Nepali, Mira Park, Helen Lew, Okjoon Kim

**Affiliations:** ^1^Department of Opthalmology, CHA Bundang Medical Center, CHA University, Seongnam, Republic of Korea; ^2^Department of Neurology, CHA Bundang Medical Center, CHA University, Seongnam, Republic of Korea

## Abstract

Adipose tissue contains abundant multipotent mesenchymal stem cells with strong proliferative and differentiating potential into adipocytes, osteocytes, and chondrocytes. However, adipose-derived mesenchymal stem cells (ASCs) showed variable characteristics based on the tissue-harvesting site. This study aimed at comparing human adipose-derived mesenchymal stem cell from the orbit (Orbital ASCs) and abdomen (Abdominal ASCs). Orbital and abdominal ASCs were isolated during an upper or lower blepharoplasty operation and liposuction, respectively. Flow cytometric analysis was done to analyze the surface antigens of ASCs, and cytokine profiles were measured using Luminex assay kit. The multilineage potential of both ASCs was investigated using Oil Red O, alizarin red, and alcian staining. Reverse transcriptase polymerase chain reaction (RT-PCR) was performed to measure mRNA levels of genes involved in these trilineage differentiations. Our results showed that both types of ASCs expressed the cell surface markers which are commonly expressed stem cells; however, orbital-ASCs showed higher expressions of CD73, CD90, CD105, and CD146 than abdominal ASCs. Unlikely, orbital-ASC expressed CD31, CD45 and HLA-DR lesser than abdominal-ASCs. Orbital ASCs secreted higher concentrations of eotaxin, fractalkine, IP-10, GRO, MCP-1, IL-6, IL-8, and RANTES but lower MIP-1*α*, FGF-2, and VEGF concentrations than abdominal-ASCs. Our result showed that orbital ASCs have higher potential towards adipogenic and osteogenic differentiation but lower tendency to chondrogenesis when compared with abdominal ASCs. In conclusion, tissue-harvesting site is a strong determinant for characterization of adipose-derived mesenchymal stem cells. Understanding defining phenotypes of such cells is useful for making suitable choices in different regenerative clinical indications.

## 1. Introduction

Mesenchymal stem cells (MSCs) are widely applied in regenerative medicine for the treatment of tissue damage due to some pathological diseases or trauma [1] and are derived from different tissues such as the bone marrow, adipose tissues [2], skin [3], muscle [4], and tendon [2]. However, clinical applicability of these MSCs for regenerative medicine has to meet the following criteria, such as abundant quantities, minimally invasive, multilineage differentiation, safe, and effective transplantation and manufacturing in accordance with current good manufacturing practice (GMP) guidelines [5]. The bone marrow is the best source of stem cell but adipose-derived mesenchymal stem cells (ASCs) can be the alternative source in the clinical field, as both show similar characteristics regarding morphology, proliferation, multipotency, and some specific markers [6, 7].

Adipose tissue can be isolated easily by various methods like blepharoplasty, levator muscle resection, and laparotomy in abundant quantity from many sites, such as the abdomen, breast, buttock, orbit, and thigh [8]. Thus, ASCs are more suitable resource for regenerative medicine applications due to its abundance and easy accessibility [9, 10]. ASCs from different tissue sites exhibit differences in characteristics; for example, traits of cells isolated from the subcutaneous adipose tissue and from the abdomen are dissimilar [11]. Specifically, stromal cells from the subcutaneous adipose tissue proliferate faster than those from the abdomen; however, no regional difference in differentiation of the cells has been found [12], while the frequency of ASC is found to be higher in the abdomen than in the thigh, or hip region [13]. Also, tissues isolated from the hip yield more stromal cells compared to those isolated from the abdomen [14]. ASCs have mesodermal origin and undergo several lineages of adipogenic, osteogenic, chondrogenic, myogenic, cardiogenic, and neurogenic differentiation [15]. Most adipose tissues have mesodermal origin but adipose tissues from the eyelid have ectodermal origin as neural crest-derived cells, such as facial muscle and cartilage [16], and also can differentiate as mesodermal lineage [17]. ASCs derived from the eyelid have been reported to have similar characteristics of the neural crest. [18].

The aim of this study is to characterize and compare human ASCs isolated form the orbit and abdominal tissues and also to compare their capacity during multilineage differentiation.

## 2. Materials and Methods

### 2.1. Subjects

The study participants were 10 healthy men aged between 35 and 55 years and included both types of tissues, orbital and abdominal from the same individual. Tissues were isolated within clinical studies approved by the institutional review board.

### 2.2. Isolation of Orbital and Abdominal Adipose-Derived Stem Cells

Orbital ASCs and abdominal ASCs were isolated following the protocol described in the previous study with minor modifications [19]. Briefly, orbital adipose tissue was obtained during an upper or lower blepharoplasty operation and abdominal adipose tissue during liposuction. The operated fat pearls were microdissected and washed in phosphate buffer solution (PBS; Welgene, Daegu, South Korea) to remove erythrocytes and microdissected and digested with 0.25% type I collagenase type II (Gibco Life Technologies, Sao Paulo, SP, Brazil) at 37°C for 60 min under constant shaking [20]. After removal of supernatant, cells were suspended and cultured in complete growth media containing Dulbecco's Modified Eagle Medium (DMEM)/F12 (Gibco; Grand Island, New York, USA) with 10% fetal bovine serum (FBS) (Gibco; Grand Island, New York, USA) and 1% penicillin-streptomycin solution (P/S) (Gibco; Grand Island, New York, USA) in 5% CO_2_ and 37°C. Cells were passaged when they reached 20–90% confluence with media changed in 2-3-day interval.

### 2.3. Flow Cytometry

Cells at passage 3 (p3) were detached by using trypsin-EDTA 0.2% (Gibco, Carlsbad, CA, USA), fixed with methanol for 10 min at −20°C, washed with 1% BSA in PBS, and then incubated with permeabilization buffer: 0.1% Triton X-100 and 1% BSA in PBS for 10 min at 4°C. Cells were incubated with antibodies raised against CD31, CD34 CD45, CD73, CD90, CD105, CD146, and HLA-DR (Santa Cruz Biotechnology, Dallas, TX, USA) on ice for 30 min. Cells were pelleted, washed, and fixed in 1% paraformaldehyde. Fluorescence-activated cell sorting (FACS) analysis was performed on BD Biosciences FacsCalibur flow cytometer (Becton Dickinson, San Jose, CA, USA) using FlowJo software for analysis.

### 2.4. Multiplex Supernatant Cytokine Assay (Luminex)

We quantified various cytokines and growth factor concentrations secreted from both types of ASCs by using Luminex multiplex assay kit (Millipore, Billerica, MA, USA) according to the manufacturer's instructions. This assay kit measured protein concentrations of chemokines: eotaxin, fractalkine, interferon-gamma inducible protein-10 (IP-10), monocyte inflammatory protein (MCP)-1, macrophage inhibitory protein (MIP)-1*α*, and RANTES; proinflammatory cytokines: granulocyte macrophage colony-stimulating factor (GM-CSF), interleukin- (IL-) 1*β*, IL-2, IL-12(p40/p70), IL-6, IL-7, IL-8, IL-15, IL-17, tissue necrosis factor- (TNF-) *α*, and interferon- (IFN-) *γ*; anti-inflammatory cytokines: IL-1RA, IL-4, IL-5, IL-10, IL-13, and IFN-*α*; and growth factors: vascular endothelial growth factor (VEGF), melanoma growth stimulatory activity (GRO), epidermal growth factor (EGF), fibroblast growth factor (FGF)-2, and platelet-derived growth factor (PDGF-AA and PDGF-BB).

### 2.5. Adipogenic Differentiation and Oil Red O Staining

Orbital and abdominal ASCs were cultured with adipogenic medium that contains 30 *μ*M indomethacin, 5 *μ*M insulin, 0.5 mM 2-isobutyl-1-methylxanthine, and 1 *μ*M dexamethasone (Sigma-Aldrich; St. Louis, MO, USA). The medium was changed every 2-3 days. At 21 days of differentiation, cells were fixed with 10% neutral formalin for 1 hour and stained with Oil Red O solution for 30 min. For quantitation of lipid vacuoles, cells were extracted with 200 *μ*l of 100% isopropanol (Merck KGaA, Darmstadt, Germany) for 15 min, and absorbance was measured using VersaMax™ Plus Rom v1.23 ELISA plate reader (Molecular Devices®, Sunnyvale, CA) at 540 nm [21].

### 2.6. Osteogenic Differentiation and Alizarin Red S Staining

Orbital and abdominal ASCs with confluent growth were cultured in a serum-free Stem Pro osteogenesis differentiation kit (Life Technologies). Cells were cultured with complete growth medium (control) or induced medium for 21 days, and the medium was changed every 2-3 days. Cells were stained with alizarin red S stain (Sigma-Aldrich; St. Louis, MO, USA), and mineralization during osteogenesis was measured quantitatively following the manufacturer's instructions. In brief, cells were incubated with 10% acetic acid for 30 min at RT, scraped and transferred to new tubes (1.5 ml), followed by heating at 85°C for 10 min, and then cooled in ice. After removal of debris by centrifugation, the supernatant was transferred to a new tube, normalized with 10% ammonium hydroxide solution, and absorbance was measured at 405 nm [22].

### 2.7. Chondrogenic Differentiation and Alcian Blue Staining

Orbital and abdominal ASCs with 80% confluency were cultured in chondrogenic differentiation kit (Life Technologies), and the medium was changed every 2-3 days. After 3 weeks, cells were stained with alcian blue stain (Sigma) according to the manufacturer's protocol. For quantitative measurement, stained cells were extracted with 6 M guanidine HCl for 2 h at RT and absorbance was measured at 650 nm [23].

### 2.8. Quantitative Real-Time Polymerase Chain Reaction (qPCR)

Total RNA was extracted from orbital and abdominal ASCs using TRIzol RNA isolation reagent, according to the manufacturer's instructions. RNA was quantified with NanoDrop 1000 (Thermo Fisher, Waltham, MA, USA). We used 1 *μ*g of RNA for cDNA synthesis with amfiSure Taq DNA polymerase (GenDEPOT, USA). We performed qPCR by using SYBER Green PCR Master Mix (Bio-Rad, USA) on CFX96 real-time PCR detection system (Bio-Rad). The primers used for qPCR are listed in Table 1. Real-time PCR reactions were determined in triplicate with initial activation at 95°C for 2 min, followed by 40 cycles: 95°C for 30 seconds, 60°C for 30 seconds, and 72°C for 20 seconds. For the cycle number at which the amount of amplified genes reached a fixed threshold, threshold cycle (Ct) was determined. Relative gene expression was calculated by normalizing the difference of genes of interest in the cycle threshold value (delta Ct) with the delta Ct value of endogenous control, 18s ribosomal RNA *(18s rRNA)*.

### 2.9. Statistics

Results are expressed as means ± SEM and analyzed using GraphPad Prism software (version 5.0, GraphPad Software, San Diego, California). The two-tailed unpaired Student *t*-test was used to analyze normally distributed data, and the Mann–Whitney test was used to analyze nonparametric data. Statistical significance was accepted for *p* value < 0.05.

## 3. Results

### 3.1. Phenotypic Characterization of Orbital and Abdominal ASCs

**Figure 1:**
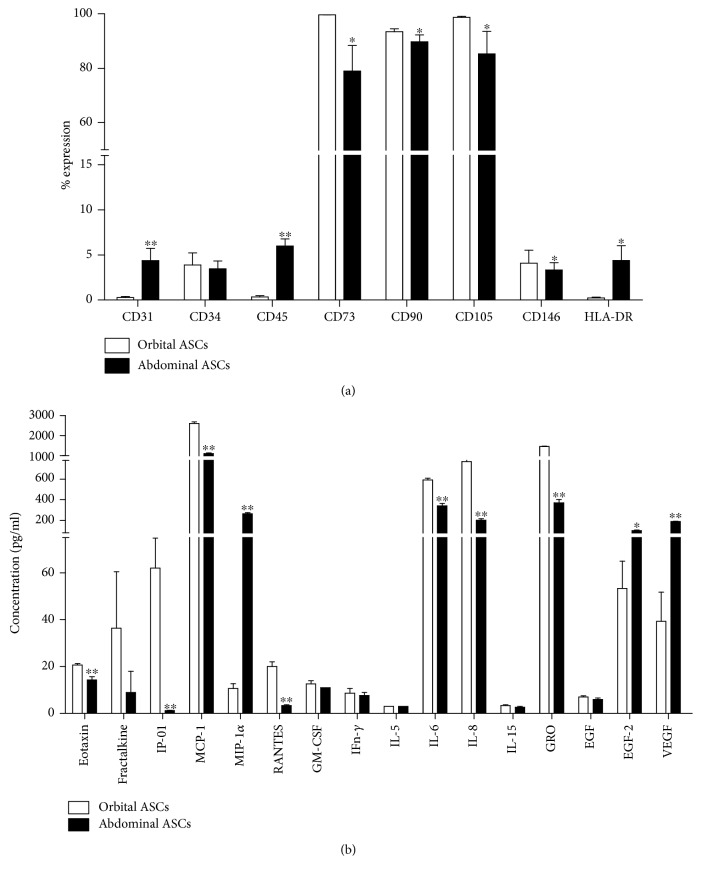
Phenotypical characterization of orbital and abdominal ASCs. (a) Flow cytometric analysis of surface markers of ASCs. (b) Luminex assay to measure secreted cytokines by both types of ASCs. Results are presented as means ± standard error mean (SEM). ^∗^*p* < 0.05 and ^∗∗^*p* < 0.01.

Flow cytometric analysis resulted that orbital ASCs expressed CD31, CD45, and HLA-DR expressions by 93.64, 94.19, and 94.40% lower than abdominal ASCs, respectively. However, both types of ASCs expressed significantly low percentage of CD34 expression which is one of the characteristic features of stem cells. The typical markers of stem cells, such as CD90, CD73, CD105, and CD146, were significantly higher in both types of ASCs; however, orbital ASCs showed these expressions higher than abdominal ASCs by 3.93, 20.73, 13.61, and 18.35%, respectively (Figure 1(a)).

### 3.2. Orbital and Abdominal ASCs Secreted Variable Levels of Cytokines

Quantitative chemokine assay showed that orbital ASCs showed higher concentrations of chemokines, such as eotaxin, fractalkine, IP-10, MCP-1, and RANTES, respectively, by 30.6, 66.2, 57.8, and 83.3% but lower concentrations of MIP-1*α* by 96% when compared with abdominal ASCs. Among proinflammatory cytokines, orbital ASCs secreted higher concentrations of IFN-*γ*, IL-5, IL-6, and IL-8 than abdominal ASCs by 11.5, 42.4, and 73.8%, respectively, whereas other proinflammatory proteins, such as TNF-*α*, IL-1*β*, IL-2, IL-12(p40/p70), and IL-17, were not detected in both types of ASCs. Furthermore, considering anti-inflammatory cytokines, both types of ASCs expressed IL-5 in similar concentrations but none of the other mentioned cytokines were detected in any supernatant. In addition, orbital ASCs secreted 14.3% higher concentrations of EGF than abdominal ASCs but FGF-2 and VEGF were lower by 47.5 and 79.2%, respectively. Other growth factors, like PDGF-AB and IGF1, were not detected in the supernatant illustrating that their concentrations were lower than the detection limit of the corresponding assays.

### 3.3. Comparison of Adipogenic Differentiation of Orbital and Abdominal ASCs

**Figure 2:**
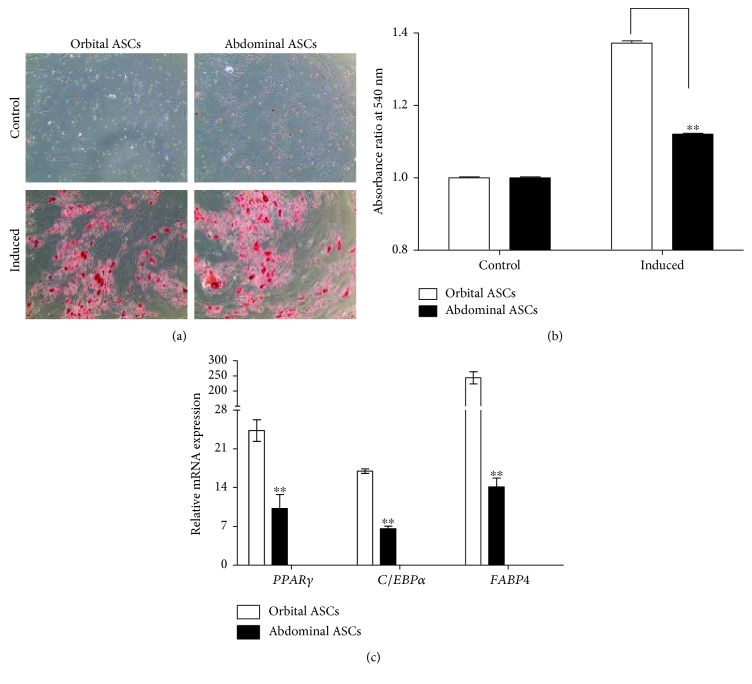
Adipogenic differentiation of orbital and abdominal ASCs towards adipogenesis. (a) Microscopic photograph of ASCs cultured in adipogenic medium for 21 days and subjected to lipid staining with Oil Red O. Control cells were grown in DMEM/F12 medium for the same period of time. (b) Quantitation analysis of lipid droplet accumulation by determining the amount of dye extracted with isopropanol and measured at absorbance 540 nm. (c) Real-time PCR analysis for genes involved in adipogenesis, such as *PPARγ*, *C/EBPα*, and *FABP4*. Results are presented as means ± standard error mean (SEM). ^∗^*p* < 0.05 and ^∗∗^*p* < 0.01.

Orbital and abdominal ASCs were induced with adipogenic medium for 21 days to examine the lipid droplets during adipogenesis and compared with noninduced cells or control (cells grown on complete growth media). Oil Red O staining results showed orange color for lipid droplets which was higher in orbital ASCs when compared with abdominal ASCs (Figures 2(a) and 2(b)). Furthermore, qPCR analysis showed that mRNA expressions of genes involved in adipogenesis, such as *PPARγ*, *E/EBPα*, and *FABP4*, were higher in orbital ASCs than in abdominal ASCs by 57.9, 61.1, and 94.2%, respectively (Figure 2(c)).

### 3.4. Comparison of Osteogenic Differentiation of Orbital and Abdominal ASCs

**Figure 3:**
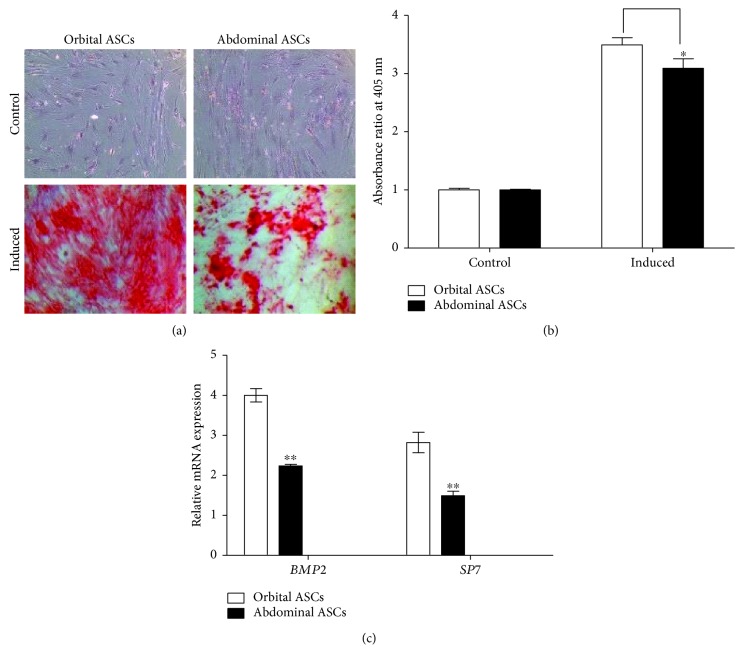
Osteogenic differentiation of orbital and abdominal ASCs towards osteogenesis. (a) Microscopic photograph of ASCs cultured in osteogenic medium for 21 days and stained with alizarin red stain. Control cells were grown in DMEM/F12 medium for the same period of time. (b) Quantitation analysis of mineralization by determining the amount of dye extracted and measured at absorbance 405 nm wavelength. (c) Real-time PCR analysis for genes involved in osteogenesis, such as *BMP2* and *SP7*. Results are presented as means ± standard error mean (SEM). ^∗^*p* < 0.05 and ^∗∗^*p* < 0.01.

Orbital and abdominal ASCs were subjected to osteogenic medium for 21 days in culture. Our result showed that both types of ASCs showed positive staining with alizarin red stain in osteogenic medium (induced) when compared to complete growth medium (control) (Figure 3(a)). The extraction of alizarin red showed the quantitative results of deposition of calcium crystal which was significantly higher in orbital ASCs by 4.9% than abdominal ASCs cultured in osteogenic medium (Figure 3(b)). Furthermore, to elucidate the genes involved in osteogenesis, qPCR analysis was performed. Our result showed that orbital ASCs showed significantly higher expressions of *BMP2* by 44.1% and *SP7* by 47.2% than abdominal ASCs (Figure 3(c)).

### 3.5. Comparison of Chondrogenic Differentiation of Orbital and Abdominal ASCs

**Figure 4:**
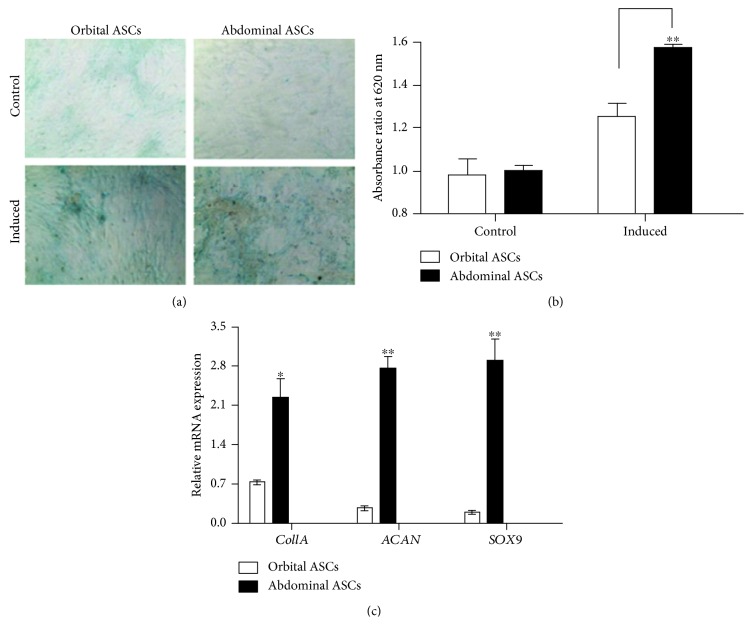
Chondrogenic differentiation of orbital and abdominal ASCs towards chondrogenesis. (a) Microscopic photograph of ASCs cultured in chondrogenic medium for 21 days and stained with alcian blue. Control cells were grown in DMEM/F12 medium for the same period of time. (b) Quantitation analysis of extracellular matrix production by determining the amount of dye extracted with 6 M guanidine HCl and measured at absorbance 620 nm wavelength. (c) Real-time PCR analysis for genes involved in chondrogenesis, such as *Col1A*, *ACAN*, and *SOX9*. Results are presented as means ± standard error or mean (SEM). ^∗^*p* < 0.05 and ^∗∗^*p* < 0.01.

After 21 days of chondrogenic induction, ASCs were stained with alcian blue stain and compared with noninduced cells. ASCs induced with chondrogenic medium showed aggregation of cells and increased extracellular matrix production which was stained positively by blue color in alcian blue staining as shown in (Figures 4(a) and 4(b)). Furthermore, to determine whether the ASCs underwent chondrogenesis, the mRNA expressions of genes involved in chondrogenesis were assessed by qPCR analysis. Our results showed that abdominal ASCs significantly expressed higher *Col1A*, *ACAN*, and *SOX*9 mRNA levels than orbital ASCs by 67.1, 90.2, and 93.3%, respectively (Figure 4(c)). Thus, our result showed that orbital ASCs were less potent to chondrogenic differentiation when compared to abdominal ASCs.

## 4. Discussion

This study aimed at comparing the characteristic alteration and differentiation capacity between adipose-derived mesenchymal stem cells (ASCs) and orbital and abdominal tissues. Though orbital and abdominal ASCs were not detached naturally but could be harvested by collagenase digestion then expanded *in vitro*, they displayed different characteristics in terms of expression profile of cytokines and surface antigens. Our study illustrates for the first time the phenotypic characteristics between orbital and abdominal ASCs along with their trilineage tendency of differentiation.

ASCs derived from any site are supposed to express mesenchymal stem cell (MSC) markers but not consistently express all the characteristics of MSC, and the profile expression changes with culture time [24]. Also, MSCs are known to have negative expressions of hematopoietic surface markers, cluster of differentiation (CD34, CD45, and HLA-DR), and endothelial marker (CD31) but high expressions of CD73, CD90, and CD105 [25, 26]. CD45 is found in hematopoietic cells and regulates cell growth, differentiation, mitotic cycle, and oncogenic transformation [27]. CD90 is a membrane-bound glycoprotein which is expressed by almost 90% of variable tissues [28], and its function is related to angiogenesis [29, 30]. Additionally, CD146, a typical pericyte marker, is reported as common surface marker of MSCs [31–34], and its expression is dependent on the donor or the cell passage number [35, 36]. The previous report showed the variation of CD90 and CD146, neuron-specific enolase (NSE), and nuclear receptor-related protein 1 (Nurr1) in ASCs derived from the eyelid and abdominal fat [37]. Our findings showed that orbital ASCs contain surface markers related to MSCs in higher levels than abdominal ASCs. In accordance with our results, ASCs contain uniform characteristic markers, positive for CD44, CD73, CD90, and CD105 and negative for CD31, CD45, and HLA-DR [31, 38].

Most chemokines, such as RANTES, MCP-1, and IP-10, attract the inflammatory cells, dendritic cells, monocytes, macrophages, and T lymphocytes, while IL-6 secretion is associated with pluripotency and immune privilege of MSCs [39, 40]. It is reported that fractalkine, a member of membrane-bound chemokines, has a role in osteoclast differentiation [41], and other chemokines, such as eotaxin and MIP-1*α*, have a role in eosinophil recruitment in MSCs [42]. In our study, orbital ASCs showed more immunological safety and higher concentration of chemokine production versus abdominal ASCs. Also, none of the anti-inflammatory markers except IL-5 was detected however; most proinflammatory mediators that were expressed by both types of ASCs were variable. ASCs are reported to be a mediator of tissue regeneration as they secrete specific soluble factors, such as VEGF and FGF [43]. Our results showed both types of ASCs secreted VEGF, EGF, and FGF-2; however, secretions of FGF-2 and VEGF are lesser by orbital ASCs versus abdominal ASCs. Thus, our study illustrates that ASCs derived from different sites have different properties in terms of inflammation and regeneration.

Adipogenic differentiation is regulated by a complex network of transcription factors that begins with increased expressions of CCAT/enhancer-binding protein (C/EBP) *β*/*γ*, which activates *PPARγ* and *C/EBPα* [44]. It is reported that *FABP4* is an adipokine with a distinct role of transcriptional and metabolic regulation in ASCs [45]. In this study, ORO staining results showed that both ASCs were induced when cultured in adipogenic medium and orbital ASCs have more adipogenic property compared to abdominal ASCs, which was ascertained by mRNA expressions of genes involved in adipogenesis. Orbital ASCs expressed higher mRNA levels of *PPARγ*, *C/EBPα*, and *FABP4* than abdominal ASCs.

Previous studies reported that *BMP2* plays an important role in cell adhesion, proliferation, and maturation of extracellular matrix during osteogenesis [46, 47]. This study revealed that orbital ASCs expressed higher *BMP2* and *SP7* mRNA levels than abdominal ASCs, illustrating that orbital ASCs are more potent to osteogenic differentiation. These increased expressions of genes are concurrent with the recent findings of genes involved in osteogenesis [48].

*SOX9* is a master regulator for the expression of major cartilage matrix protein type II collagen [49]. In our study, abdominal ASCs showed increased *Col1A*, *ACAN*, and *SOX9* mRNA expressions than orbital ASCs. In accordance with this result, the recent study presented that osteoarthritic patients showed increased expressions of these genes during the chondrogenic differentiation [50].

The present study has some limitations that should be noted. Firstly, we did not determine the mRNA expressions during the early stage of differentiation of ASCs, and secondly, protein levels of signaling mechanisms involved in trilineage differentiations were not investigated. To the best of our knowledge, this is the first comparative study of ASCs derived from orbital and abdominal fat tissues with their trilineage capabilities of differentiation. Collectively, although isolated from similar adipose tissues, both types of ASCs displayed many contrasting characteristics in terms of surface markers and cytokine release. In addition, orbital ASCs have more capabilities towards adipogenesis and osteogenesis, but less tendency to chondrogenic differentiation when compared to abdominal ASCs. Further studies on the mechanism of trilineage differentiation need to be elucidated. Understanding defining phenotypes of such cells is useful for making suitable choices in different regenerative clinical indications like cell-based therapy and tissue engineering.

## Figures and Tables

**Table 1:** Lists of human primer sequences used for reverse transcription polymerase chain reaction (RT-PCR).

Primers	Forward sequences	Reverse sequences
*PPARγ*	ATTGACCCAGAAAGCGATTC	ATTGACCCAGAAAGCGATTC
*C/EBPα*	GGGTCTGAGACTCCCTTTCCTT	CTCATTGGTCCCCCAGGAT
*FABP4*	AACCTTAGATGGGGGTGTCCTG	TCGTGGAAGTGACGCCTTTC
*BMP2*	TTTGGACACCAGGTTGGTGAA	ACGAATCCATGGTTGGCGT
*SP7*	GCACAAACATGGCCAGATTC	AGA AATCTACGAGCAAGGTC
*COl1A*	TCCTGCCGATGTCGCTATC	CAAGTTCCGGTGTGACTCGTG
*ACAN*	CCTCCCCTTCACGTGTAAAA	GCTCCGCTTCTGTAGTCTGC
*SOX9*	TACCCGCACTTGCACAAC	TCTCGCTCTCGTTCAGAAGTC
*18*s *rRNA*	TGAGAAACGGCTACCACATC	ACTACGAGCTTTTTAACTGC

*PPARγ*: peroxisome proliferator-activated receptor gamma; *C/EBPα*: CCAT/enhancer-binding protein alpha; *FABP4*: fatty acid-binding protein 4; *BMP2*: bone morphogenetic protein 2; *SP7*: Osterix; *COl1A*: collagen type I; *ACAN*: aggrecan; *SOX9*: Sry-type high-mobility group (HMG) box 9; *18s rRNA*: 18s ribosomal RNA.

## Data Availability

The human adipose tissues were supplied by Bundang CHA Medical Center and approved by the institutional review board. Sequences of primers used in our study are available with accession number and can be provided upon request. All data are provided in full in Results of our manuscript, and the necessary detail can be provided by the corresponding author under request.
